# Is admission of hematologic malignancies in the ICU justified?

**DOI:** 10.1186/cc14618

**Published:** 2015-03-16

**Authors:** VG Gasparovic, MM Grgic Medic, IG Gornik

**Affiliations:** 1 KBC Zagreb, Croatia

## Introduction

The admission of malignant hematology patients to the ICU is combined with poor prognosis [1]. A young population on one side and serious prognosis on the other are the main characteristics. Do we help? We are analyzing continuously clinical characteristics, treatment, and outcomes of critically ill patients with hematologic malignancies admitted to the medical ICU to identify predictors of adverse outcome [2].

## Methods

Demographic characteristics, hematologic diagnosis, reasons for ICU admission, transplant status, the presence of neutropenia, and APACHE II and SOFA scores were analyzed. Predictors of ICU mortality were evaluated using univariate analysis.

## Results

There was a total of 194 patients (103 male), APACHE II score by admission was 27 ± 8, SOFA 9 ± 3. Acute leukemia (L) in 81 patients (41.8%), chronic L in 19 patients (9.8%), lymphoma in 58 patients (29.9%), and multiple myeloma in 28 patients (14.4%) were the etiology. Respiratory insufficiency, hemodynamic instability, AKI and CNS disturbances were responsible for the admission of 169 patients (87.1%) from the hematology ward to the ICU. In total, 127 patients (59.7%) were mechanically ventilated; 93 required invasive mechanical ventilation (MV). Non-invasive ventilation started in 34 patients and was successful in 14 (6.5%). The ICU mortality rate was 104 patients (53.6%), and the mortality of MV patients was 98 (77.2%). Need for vasopressors at admission, MV, neutropenia, and APACHE II and SOFA scores were identified as independent predictors of fatal outcome. Overall mortality of admitted patients was 53.6% (104 patients), and in ventilated patients was 77.2% (98 patients).

## Conclusion

The ICU mortality of critically ill patients with HM is high, particularly in the group on MV. Different factors were independent predictors of mortality, but 46.4% of admitted patients survived because of adequate support possibilities and were transferred back to hematology ward. The ICU admission with organ support is according

## Results

important for life saving in this extremely high-risk patient group.
